# Immuno-Dipstick for *Colletotrichum gloeosporioides* Detection: Towards On-Farm Application

**DOI:** 10.3390/bios12020049

**Published:** 2022-01-18

**Authors:** Fifame Auriane Oussou-Azo, Taiki Futagami, Mun’delanji Catherine M. Vestergaard

**Affiliations:** 1 United Graduate School of Agricultural Sciences, Kagoshima University, Kagoshima 890-0065, Japan; oauriane1@gmail.com (F.A.O.-A.); futagami@agri.kagoshima-u.ac.jp (T.F.); 2 Faculty of Agriculture, Kagoshima University, 1-21-24 Korimoto, Kagoshima 890-0065, Japan

**Keywords:** *Colletotrichum gloeosporioides*, anthracnose, immuno-dipstick, loop-mediated amplification (LAMP), simplified DNA extraction, point-of-need molecular diagnostic

## Abstract

Early and quick detection of pathogens are crucial for managing the spread of infections in the biomedical, biosafety, food, and agricultural fields. While molecular diagnostics can offer the specificity and reliability in acute infectious diseases, detection of pathogens is often slowed down by the current benchtop molecular diagnoses, which are time consuming, labor intensive, and lack the mobility for application at the point-of-need. In this work, we developed a complete on-farm use detection protocol for the plant-devastating anthracnose agent: *Colletotrichum gloeosporioides*. Our methods combined a simplified DNA extraction on paper that is compatible with loop-mediated isothermal amplification (LAMP), coupled with paper-based immunoassay lateral flow sensing. Our results offer simple, quick, easy, and a minimally instrumented toolkit for *Colletotrichum gloeosporioides* detection. This scalable and adaptable platform is a valuable alternative to traditional sensing systems towards on-the-go pathogen detection in food and agriculture, biomedical, and other fields.

## 1. Introduction

Anthracnose is a serious fungal disease that many affects economically important crops worldwide including cereals, legumes, vegetables, and fruits [1]. The causal agent is *Colletotrichum gloeosporioides* (*C. gloeosp*), one of the most destructive plant pathogens [2]. It is reported as one of the most widespread, severe, and economically important diseases for tropical and subtropical crops [3,4,5,6]. Members of the genus *Colletotrichum* have a wide distribution worldwide, and affect a wide variety of crops including cashew, bean, chili, passion fruits, mango, avocado, apple, coffee, and strawberry plants [1,2,7,8,9,10,11]. The disease’s symptoms can be seen on leaves and young fruits [12]. The pathogen attacks immature fruits and produces necrotic lesions [13]. The infection is seed borne, soil borne, water borne, and airborne. It leads to a significant reduction in yield and subsequent economic loss due to the fact that it attacks crops both pre- and post-harvest [7,8,9,14].

Jaiza and colleagues reported that anthracnose infection may occur throughout the year, but large damages take place during the rainy season [6]. While environmental factors play an important role in the spread of the disease, early detection of the pathogen is of the utmost importance. This would enable early intervention and management control, thus limiting the production and economic losses. One of the profound challenges is detecting the pathogen on asymptomatic plants. Indeed, while anthracnose disease can be clearly identified by morphological symptoms, sometimes the symptoms are masked as the disease survives in the form of latent infection in the absence of a congenial environment [15,16]. In such cases, symptom-based detection of the pathogen may not be accurate and reliable, early detection with a fast, sensitive, and specific diagnostic test and timely intervention are crucial for preventing the disease’s spread and outbreaks. Another outstanding challenge is on-field detection. Current DNA detection methods mainly employ polymerase chain reaction (PCR) and gel electrophoresis in the laboratory, and these techniques cannot be utilized on-field [17].

Over the past decade, isothermal nucleic acid amplification methods have been developed to overcome the complex instrumentation, time, and training required for PCR-based analysis [18,19]. Since its discovery, loop-mediated isothermal amplification (LAMP) technology has played an important role in molecular diagnostics. LAMP is a novel nucleic acid amplification assay that relies on auto-cycling strand displacement DNA synthesis catalyzed by *Bst* DNA polymerase [20]. It has been reported that among numerus nucleic acid amplification assays, LAMP stands out in terms of sample-to-answer time, sensitivity, specificity, cost, robustness, and accessibility, making it ideal for on-field use [20]. Indeed, the most common diagnostic method (PCR) is used in conjunction with gel electrophoresis that requires highly toxic ethidium bromide, and it requires costly thermocyclers and tedious technical optimization of cycling conditions. In contrast, a positive LAMP reaction can alternatively be observed visually based on either the turbidity of the magnesium pyrophosphate solution, or the use of calcein–manganese labeling techniques [21,22]. Disposable and simple-to-use paper-based detection tools used in conjunction with LAMP are being exploited for their versatility and potential for utilization on-field. They include lateral flow dipsticks (LFDs) [23]. Studies by Ranaganathan et al. [24] and Safenkova et al. [25] have described lateral flow as point-of-care, rapid response, easy storage, user-friendly design, low cost, and on site application tests that allow users to detect a targeted analyte’s presence quickly without heavy and complex instruments. Such rapid observation of the results directly by the naked eye will ensure the convenience of performing bioassays in the field at the point-of-care and in resource-limited settings.

The lateral flow test strips are based on biosensor technology. Briefly, a biosensor is an analytical device that is based on a biological molecule as its recognition element. The biomolecule is chosen for its specificity (affinity or catalytic) towards the target analyte. The most commonly used natural biomolecules are DNA and antibodies (immunoglobulins) because of the natural intrinsic specificity towards their targets, binding with high affinity using the Crick–Watson base-paring and antibody–antigen interaction, respectively. The binding or interaction event is detected/measured using a transducer. This can be electrochemical, optical, piezoelectric, etc., depending on the sensing platform [26]. Most paper-based biosensors use noble metals (Au and/or Ag) as molecular labels to enable visual detection of the binding events [27,28]. When the target analyte is an antigen (e.g., protein), the most commonly used recognition molecule on the paper surface is an antibody, using a sandwich format [29,30,31]. However, when the target analyte is DNA, as was the case in our study, an antibody–DNA–antibody sandwiched format was used as a molecular recognition element. In this case, molecular labels, for example, haptens, such as biotin, digoxigenin, carboxyfluorescein, fluorescein isothiocyanate (FITC), and dinitrophenol, and the corresponding anti-hapten antibodies were employed [32,33,34,35]. One or more antibodies are conjugated with the noble metal nanoparticles (NPs). The special physical properties of metal NPs (e.g., plasmonic resonance) are dependent on the size and shape of the NPs. Briefly, as the size of a metal structure decreases from the bulk scale to the nano-scale (<100 nm), the movement of electrons through the internal metal framework becomes restricted. Consequently, metal NPs display extinction bands in their UV–Vis spectra when the incident light resonates with the conduction band electrons at their surfaces. These charge density oscillations are called longitudinal surface plasmonic resonance (LSPR). The excitation of LSPR by light at an incident wavelength, where resonance would occur, results in the appearance of intense surface plasmon (SP) absorption bands. The type, size, and shape of the NPs, and their distribution affect the intensity and position of the SP absorption [36]. AuNPs with a size range between 10 and 40 nm have been used as immunolabels for visual detection of *Escherichia coli*, *Pseudomonas aeruginosa*, influenza virus (A/H1N1), and cyprinid herpes virus-3 DNA amplicons [33,35,37,38].

To date, there are only a few reports on the rapid detection of *Colletotrichum* species in infected plants using LAMP [16,39], especially towards detection on-field. To the best of our knowledge, there is no report of paper-based detection of this crop’s devastating pathogen. In the present study, we developed a combination of methods with the aim of on-farm testing: (i) quick, simple, and visual detection of *C. gloeosp.* on a paper biosensor; (ii) simplified on-paper DNA extraction compatible with both PCR and LAMP amplifications. All methods require minimal instrumentation, which can be made portable. Using these proof-of-principle methods, we successfully detected *C. gloeosp.* for strawberries in Japan. Selectivity of the biosensor was tested against *C. theobromicola* and *C. candidum*, closely related pathogens of *C. gloeosp.*, and *Aspergillus fumigatus* (*A. fum.*, a globally distributed fungus found in soil and decaying vegetation.

## 2. Materials and Methods

### 2.1. Chemicals

*C. gloeosp.* (strain QPg961, GenBank accession no. EU200455), *C. theobromicola* (*C. theo.*), and *C. candidum* (*C. cand.*) were a gift from the Laboratory of Plant Pathology, Faculty of Agriculture, Kagoshima University, Kagoshima, Japan. *A. fum.* was also gifted by the Education and Research Center for Fermentation Studies, Faculty of Agriculture, Kagoshima University, Kagoshima, Japan. Potato dextrose broth (PDB, P6685-250G), agar (CAS: 9002-18-0), bovine serum albumin (BSA, CAS: 9048-46-8), casein (CAS: 9000-71-9), Tween-20 (polyoxyethylene-20-sorbitan monolaurate, CAS: 9005-64-5), sucrose (CAS: 57-60-1), 2-propanol (CAS: 67-63-0), polyethylene glycol (PEG, CAS: 25322-68-3), Tris-HCl (CAS: 1185-53-1), sodium azide (NaN_3_, CAS: 26628-22-8), potassium dihydrogen phosphate (KH_2_PO_4_, CAS: 7778-77-0), dipotassium hydrogen phosphate (K_2_HPO_4_, CAS: 7758-11-4), sodium chloride (NaCl, CAS: 7647-14-5), boric acid (CAS: 10043-35-3), sodium dodecyl sulphate (SDS, CAS: 151-21-3), and magnesium chloride (MgCl_2_, CAS: 7786-30-3) were purchased from Sigma–Aldrich Co., Inc., (Tokyo, Japan). A colloidal gold solution (AuNPs, EM.GC30), anti-fluorescein isothiocyanate (anti-FITC) antibody, anti-biotin antibody, and rabbit anti-goat IgG secondary antibody were obtained from Funakoshi (Tokyo, Japan). The plastic backing (HF000MC100) and the absorption pad (CFSP203000) were acquired from Millipore (Burlington, MA, USA). The nitrocellulose membrane (UniStart CN140) was obtained from Sartorius Stedim Biotech (Goettingen, city, Germany). All other materials were used without further purification. Deionized water (Milli-Q water) with a resistivity of 18.2 MΩ·cm was obtained from a Millipore Synergy UV Water Purification System (Millipore, Bedford, MA, USA). All labeled and non-labeled oligonucleotides were synthesized by FASMAC (Kanagawa, Japan).

### 2.2. Preparation, Optimization, and Stability of AuNPs–Antibody Conjugates

In this current work, anti-biotin secondary antibody was conjugated with AuNPs (30 nm diameter) based on previous work that used 40 nm AuNPs [34,40] as a molecular label for DNA detection. Since the optical properties of AuNPs depend on several parameters, including size [36], optimization was required. Several parameters are crucial for determining the stability of the antibody–NPs conjugates and their capacity to assure a visual detection. These include type of buffers and buffer concentrations, temperature and pH, concentration of the NP solution, and the ratio of secondary antibody to the NPs label. Moreover, the experimentation process was completely empirical and depended on the reagents and the features of the membranes used [41,42,43,44,45]. In our work, first, we tested three different buffer concentrations at a fixed pH and temperature. A mixture of the AuNPs and the anti-biotin antibody prepared in each buffer were incubated for 10 min at room temperature (RT), the most stable buffer mixture was retained to be used in the further steps (Figure S1, Supplementary Materials (SM)). Next, different concentrations of AuNPs solution were evaluated for the visual detection test of the strip (Table S1 in SM). The main parameter evaluated in our optimization process was the color of the AuNPs, one of the most important features of the noble metal nanoparticles that reflect their stability and their functionalized state [41,46,47,48]. An optimal of 0.02% (*w*/*v*) of AuNPs colloidal solution was then considered. After, the ratio of anti-biotin antibody to AuNPs was evaluated. The optimal concentration of the anti-biotin antibody was chosen after the functionalization process was completed with different concentration of the antibody to a fixed volume and concentration of nanoparticles (Figure S2 in SM). Finally, conjugation reactions were conducted by adding anti-biotin antibody into 1 mL of AuNPs solution (0.02% (*w*/*v*)) and mixed immediately. The mixture was incubated at RT for 10 min for the immobilization of the antibodies onto the nanoparticles’ surface. Then, 1% (*w*/*v*) PEG and 10% (*w*/*v*) BSA prepared in 50 mM KH_2_PO_4_ (pH 7.5 and 9.0, respectively) were added to the anti-biotin–AuNPs conjugates solution. This was followed by centrifugation to separate bound and unbound antibodies and blocking using preserving solution (1% (*w*/*v*) BSA, 0.05% (*w*/*v*) PEG 20000, 0.1% (*w*/*v*) NaN_3_, and 150 mM NaCl, all prepared in 20 mM Tris-HCl buffer, pH 8.2). After pulse-sonication, the anti-biotin–AuNPs conjugates were stored at 4 °C until needed.

### 2.3. Fabrication of an Immuno-Dipstick

**Figure 1:**
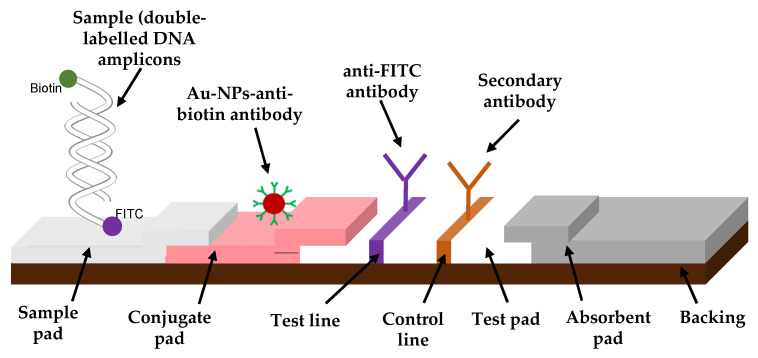
Schematic diagram of the fabricated immuno-dipstick.

The test strip consisted of mainly two components assembled on a plastic backing sheet. After an optimization of their concentration and volume (Figure S2 in SM), anti-FITC antibody solution was prepared to 0.87 mg/mL by diluting with 50 µL of 20% (*w*/*v*) sucrose solution prepared in 50 mM KH_2_PO_4_ buffer (pH 7.5) and 50 µL of 2-propanol. After, 40 µL of the anti-mouse IgG antibody was mixed with both 60 µL of 2-propanol and 1100 µL of 50 mM KH_2_PO_4_ buffer (pH 7.5). The nitrocellulose membrane, also known as a test pad (Figure 1), was prepared by immobilizing manually the anti-FITC antibody solution and the anti-mouse IgG solution, respectively, to the test and the control dots. After drying the membrane for at least 2 h at RT, the nitrocellulose membrane was blocked for non-specific protein adsorption by immersing it in 50 mM boric acid solution containing 0.5% (*w*/*v*) casein (pH 8.5) for 30 min at RT. Then, the blocked membrane was washed with 50 mM phosphate buffer saline (pH 7.5) containing 0.01% (*w*/*v*) SDS for 30 min at RT. After allowing the membrane to dry overnight at RT, it was pasted on the backing sheet with the absorbent pad on one end. Finally, the membrane was manually cut into 4 mm wide strips and stored in a desiccated container at 4 °C. All experiments were conducted at approximately 45–47% humidity level.

### 2.4. Pathogen Sample Preparation

Fungal isolates were first routinely cultured on PDA in agar plates to build a stock of culture. Agar plates are incubated at 28 °C for at least 7 d until a good culture was obtained. Once the fungi were at an active growth stage, agar blocks were cut and transferred into PDB. After incubation, fungal mycelia were collected using a filter paper, aliquoted, and stored at –20 °C for further use.

After cultivation, genomic DNA of the *Colletotrichum* spp. were extracted from the fungi mycelia using MACHEREY-NAGEL DNA extraction kit (MARCHEREY-NAGEL GmbH & Co. KG, Düren, Germany) according to the manufacturer’s instructions. Thereafter, DNA was eluted and stored at –20 °C. DNA concentration was determined using a Nanodrop 8000 spectrophotometer (Thermo Fisher Scientific, Tokyo, Japan. The Cg/f Int1 and ITS4 primers previously reported by MacKenzie et al. [49] and Katoh et al. [16] were used for the conventional PCR. DNA amplification was conducted according to the supplier’s standard protocol. Briefly, PCR reaction was performed with initial denaturation at 94 °C for 2 min, followed by 30 cycles of 94 °C for 30 s, 55 °C for 30 s, 72 °C for 1 min, and a final extension of 72 °C for 7 min. A negative control without adding DNA (no template control = NTC) was similarly prepared. The amplification was confirmed by electrophoresis on 2% (*w*/*v*) agarose gel stained with GelRed^TM^ Nucleic Acid Stain (Nacalai Tesque, Inc., Kyoto, Japan), and visualized on a UV transilluminator (UVP, LLC, Upland, CA, USA).

### 2.5. Pathogen DNA Detection at Immuno-Dipstick

For detecting *C. gloeosp.* with the fabricated paper-based lateral flow biosensor, 40 µL of double-labeled double-stranded DNA amplicons were mixed with 4 µL of anti-biotin antibody–AuNPs conjugates. The mixture was quickly mixed at RT before dipping the lateral flow strip into the conjugate solution.

In order to test the selectivity of the lateral flow biosensor, the assay was carried out with other species of *Colletotrichum* and *A. fum.*. In addition, the fabricated sensor was subjected to 10-fold serial diluted DNA amplicons to evaluate its sensitivity. No template control (NTC) was used as a negative control. The amplicons were analyzed using conventional agarose gel electrophoresis to confirm DNA amplification.

### 2.6. Simplified DNA Extraction on Paper

In order to overcome one of the biggest challenges of biosensors for DNA detection on-field, which is DNA extraction, we developed a simplified DNA extraction method that could be packaged as a kit. Current extraction methods involve instrumentation that cannot easily or readily be used on-field, such as centrifuges and vortex mixers as well as a number of extraction steps and reagents. Briefly, DNA extraction procedure involved breaking down of the cell wall either by mechanical/physical process such as being ground or using enzymatic degradation [17,50,51]. The next step involves lysis of the cytoplasmic membrane as well as nuclear membrane using lysis buffer to release the DNA. Each of these steps is followed by centrifugation, filtering, and washing procedures in order to remove co-extractants (e.g., proteins). In our work, we formulated and tested four different mixtures of reagents as potential all-in-one lysis + wash buffer (Table S1). These were used in conjunction with cellulose paper as a substrate for “catching” the released DNA [52,53]. In addition, we replaced the centrifuge with an ultrasonicator with the aim of replacing this electric ultrasonicator with a portable battery-operated one for on-field use.

DNA was extracted as follows. Onto 50 mg of fungal mycelium, 300 µL of extraction buffer were added. The mixture was then ultrasonicated for 30 s followed by incubation at 60 °C for 10 min in order to lyse the cells. For the extraction methods A and B, Tween-20 was added to the mixture after 7 min of incubation, and incubation was continued for another 3 min. Whatman grade 1 filter paper, cut in 4 × 10 mm^2^ strips, was inserted into each tube containing the cells lysate for about 1 min to bind the DNA. Immediately after, each paper strip with the bound nucleic acid was transferred into another tube containing 700 µL of the suitable wash buffer (see Table S2, SM). The strips were carefully and quickly dipped into the wash buffer in order to eliminate debris, contaminants and possible amplification inhibitors. Last, the DNA was eluted into 25 µL of Milli-Q. The genomic DNA extraction procedure was conducted in triplicate for all the extraction methods tested.

### 2.7. Quantitative PCR (qPCR): Evaluation of Simplified DNA Extraction Protocol

To evaluate the DNA extraction efficiency, the extracted samples were subjected to quantitative PCR (qPCR) with Cg/f Int1 and ITS2 primers pair [49]. The amplification was carried out in a final volume of 25 µL containing 1×TB Green *Premix Ex Taq* II (Tli RNaseH Plus) (Takara Bio, Shiga, Japan), 0.4 µM of each primer, and 1 µL of DNA template. The reaction was performed using Takara Thermal Dice Real-Time System (Takara Bio) with a cycling condition of initial denaturation at 95 °C for 30 s, followed by 40 cycles at 95 °C for 5 s and 60 °C for 30 s. Amplicons of *C. gloeosp.* genomic DNA with ITS1 and ITS4 primer pair (4.0 × 10^3^, 4.0 × 10^4^, 4.0 × 10^5^, 4.0 × 10^6^, and 4.0 × 10^7^ copies) was used as the standard DNA for the absolute quantification.

To continue with the elimination of dangerous chemicals and cumbersome and expensive instrumentation, the same purified DNA obtained from our methods were amplified using LAMP. The LAMP procedure, being isothermal, is simpler than PCR and requires no cumbersome equipment. The primers used to perform the assay (Table 1) targeted the region from the gene encoding 5.8S ribosomal RNA to the internal transcribed spacer [16]. The reaction was carried out in a final volume of 25 µL reaction mixture containing 12.5 µL of 2×reaction mixture (40 mM of Tris buffer (pH 8.8), 20 mM of KCl, 16 mM of MgSO_4_, 20 mM of (NH_4_)_2_SO_4_, 0.2% (*v*/*v*) of Tween-20, 1.6 M of Betaine, and 2.8 mM of dNTPs) (EIKEN CHEMICAL Co., Ltd., Tochigi, Japan), 40 pM of each inner primers (i.e., FIP and BIP), 5 pM of each outer primers (i.e., F3 and B3), 1 µL of *Bst* DNA polymerase (EIKEN CHEMICAL Co., Ltd., Tochigi, Japan), 1 µL of fluorescent detection reagent (EIKEN CHEMICAL Co., Ltd., Tochigi, Japan), and 2 µL of DNA template. The reaction was carried out at 65 °C for 60 min followed by termination at 80 °C for 5 min. The LAMP products were visualized on a transilluminator.

## 3. Results

### 3.1. Detection of Fungal Pathogens Using Conventional Methods

**Figure 2:**
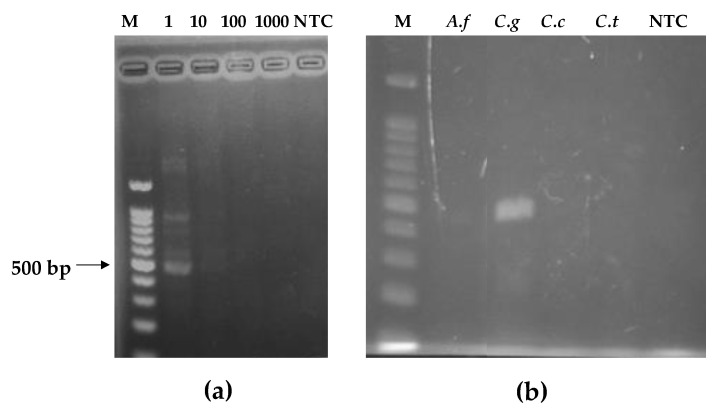
Electropherogram of PCR-amplified fungal DNA: (**a**) lane M—100 bp DNA marker; lane 1, clear visualization of *C. gloeosp*; lanes 10, 100, and 1000, *C. gloeosporioides* DNA diluted 10, 100, and 1000 times, respectively; lane NTC, no-template control; (**b**) lane M—100 bp DNA marker; lane *A.f* = *Aspergillus fumigatus*; lane *C.g* = *C. gloeosporioides*; lane *C.c* = *C. candidum*; lane *C.t* = *C. theobromicola*.

Following the pathogen culture and DNA extraction protocols (Section 2.6), the DNA of *C. gloeosp.* was quantified using a Nanodrop 8000 spectrophotometer (Thermo Fisher Scientific, Tokyo, Japan). DNA yields above 12.5 ng/µL were obtained. This analysis was important in order to have a reference for the sensitivity test and to obtain the limit of detection of the fabricated lateral flow immunosensor. Thereafter, the DNA was amplified by conventional PCR and the amplicons confirmed by gel electrophoresis. As shown in Figure 2, the clear appearance of a band at ~576 bp on the electropherogram confirmed the specificity of the primers towards our target pathogen, *C. gloeosp*. Absence of amplification was observed for all other pathogens. The 100- and 1000-fold dilution of *C. gloeosp.* yielded no amplification band. Thus, the sensitivity of this method, gave a limit of detection at 1.3 ng/µL.

### 3.2. Optimization and Stability of AuNPs–Antibody Conjugates

AuNPs show specific changes in their absorbance responses in the visible region of the spectrum upon binding with various molecules such as nucleic acids or proteins. Those changes are directly related to the sol particles, leading to a noticeable color change of the colloidal solution. In the current work, we optimized the conditions of the bioconjugation of AuNPs by testing and evaluating suitable conditions: buffer, pH and temperature, AuNPs concentration, anti-biotin-antibody-to-AuNPs ratio, concentration and volume of the immobilized antibodies as described in Section 2.2. The results showed that the combination of those different parameters assured the dispersion and stability over the time of the AuNPs conjugated with anti-biotin antibody (Figure S1 and Table S1 in SM). Thus, we used the final protocol (Section 2.2 and Section 2.3) in all our experiments thereafter. Therefore, conjugation reactions were conducted by adding anti-biotin antibody into 1 mL of AuNPs solution (0.02% (*w*/*v*)) and mixed immediately. The mixture was incubated at RT for 10 min for the immobilization of the antibodies onto the nanoparticles surface. Then, 1% (*w*/*v*) PEG and 10% (*w*/*v*) BSA prepared in 50 mM KH_2_PO_4_ (pH 7.5 and 9.0, respectively) were added to the anti-biotin–AuNPs conjugates solution. This was followed by centrifugation to separate bound and unbound antibodies, blocking using preserving solution (1% (*w*/*v*) BSA, 0.05% (*w*/*v*) PEG 20000, 0.1% (*w*/*v*) NaN_3_, and 150 mM NaCl, all prepared in 20 mM Tris-HCl buffer, pH 8.2). After pulse-sonication, the anti-biotin antibody–AuNPs conjugates were stored at 4 °C before being use.

### 3.3. Detection of C. gloeosporioides DNA at Fabricated Immuno-Dipstick

*C. gloeosp.* was successfully cultured the nucleic acid extracted and amplified it. After that initial validation using gel electrophoresis, *C. gloeosp.* doubled-labeled doubled-stranded amplicons rapid detections were evaluated by our fabricated LFD. One of the tails of the DNA amplicons with a biotin label formed a complex with the anti-biotin antibody–AuNPs, and this complex flowed along the nitrocellulose membrane by capillary action, towards the test dot. Here, it interacted with the immobilized anti-FITC antibody complementary to the other end of the PCR products with the FITC label. Excess AuNPs conjugates unbound to the amplicons flowed past the test dot and were captured at the control dot by the secondary IgG antibody. A positive (red dot) on the control line serves to confirm proper operational function of the dipstick. In the case of a negative result, no double-labeled double-stranded DNA amplicons are available to bind either the anti-biotin antibody or the anti-FITC antibody. The absorbent pad functioned as a wick to maintain the flow rate and direction, preventing any backflow of the reaction mixture.

**Figure 3:**
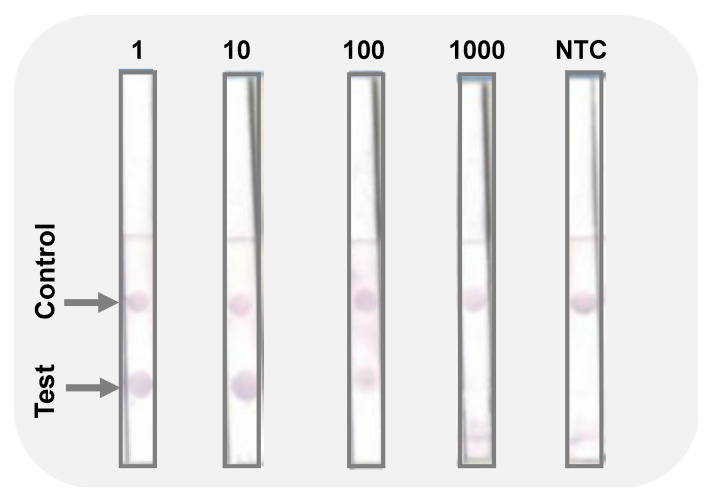
Images of the immuno-dipstick for detection of *C. gloeosporioides* DNA without dilution (1); diluted 10-fold (10); diluted 100-fold (100); 1000-fold (1000); no template control (NTC). Test and control dots are indicated by arrows.

As presented in the Figure 3, the assay was able to detect the target fungus. The control sample with no DNA present (NTC) had no dot on a test area, indicating absence of a false positive. This is important, especially when dealing with crude DNA extracts from cultured fungal mycelia. Furthermore, the fabricated biosensor was able to detect the target pathogen when the initial DNA was diluted 10 and 100 times (Figure 3), while the PCR amplification only showed amplicons in the 10 times dilution template (Figure 2a).

### 3.4. Specificity of Fabricated Immuno-Dipstick

**Figure 4:**
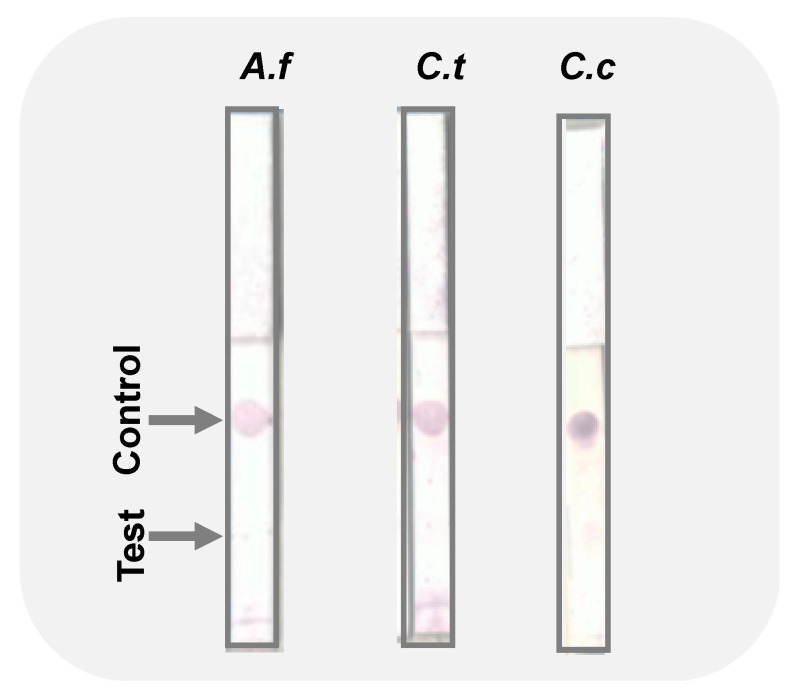
Images of the immuno-dipstick specificity test against *Aspergillus fumigatus* (*A.f*), *C. theobromicola* (*C.t*), and *C. candidum* (*C.c*). Test and control dots are indicated by arrows.

We tested the specificity of the immuno-dipstick against similar fungal genera. Since it is not uncommon to have several pathogens attack crops, it is important to be able to distinguish which pathogen(s) are present in order to institute appropriate control protocols. The analytical specificity of our lateral flow assay was assessed against *A. fum.*, and the result was negative as shown in the Figure 4. Furthermore, our fabricated sensor showed specificity against *C. theo.* and *C. cand.* as indicated by the lack of color on the test dots.

### 3.5. Simplified DNA Extraction Protocol for On-farm Use

**Figure 5:**
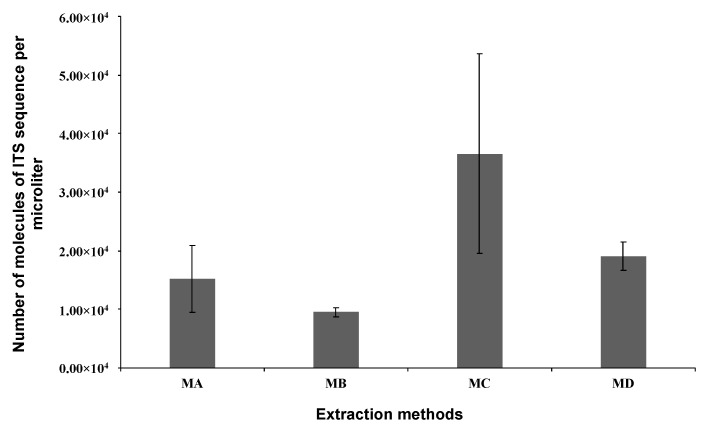
Absolute quantification of *C. gloeosporioides* DNA purified using four in-house developed simplified DNA extraction methods (MA, MB, MC, and MD; details in Table S1). Paper strips were used to purify the DNA from the samples and elute it into 25 µL Milli-Q. Purification was performed in triplicate (*n =* 3), and qPCR quantification values were used to calculate the initial concentration of ITS sequence in each amplification reaction. All bar graphs represent the mean of three replicates, and error bars represent the STD.

The DNA extraction procedures were carried out using the in-house developed lysis and wash buffers (Section 2.6, Table S1 in SM) in conjunction with cellulose matrix onto which DNA is known to adsorb to but not too strongly [52,53]. The extracted DNA was subsequently submitted to the qPCR for a qualitative and quantitative evaluation. All analyses were conducted in triplicates, and the results are shown in Figure 5. The results clearly show that all the extracted DNA was as pure as needed for a sensitive assay such as qPCR. In addition, the absolute quantification method revealed that Method C yielded the highest amount of DNA with a mean value of 3.7 × 10^4^ molecules of ITS sequence per μL of sample. Less DNA was purified with the method B, averaging at 9.5 × 10^3^ molecules of ITS sequence per μL of sample. Although not statistically different, Tween-20-containing lysis yielded less DNA than those that did not contain Tween-20. It can also be observed that wash buffers containing MgCl_2_ produced more purified DNA than those without.

**Figure 6:**
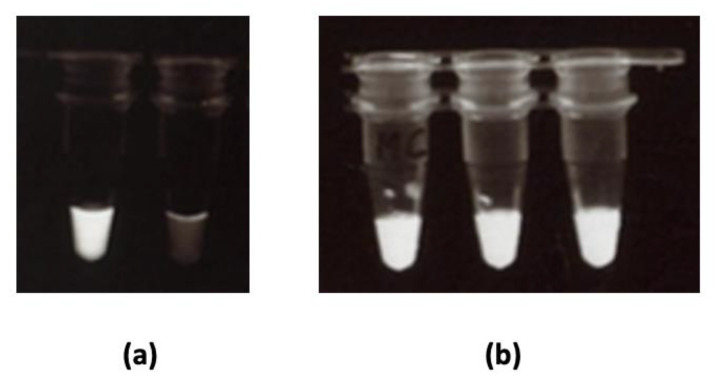
Loop-mediated isothermal amplification (LAMP) of *C. gloeosporioides* DNA extracted using (**a**) **a** conventional extraction kit: white tube had *C. gloeosporioides* DNA extract, clear tube had no DNA (negative control); (**b**) in-house simplified method C for *C. gloeosporioides* (*n* = 3). Fluorescence (milky white) indicated successful amplification.

Furthermore, we conducted a LAMP-based assay on the extracted DNA in order to confirm amplification. We used LAMP (not PCR) because we plan to further extend this work and use LAMP in conjunction with the immuno-dipstick for on-field pathogen detection. Since LAMP is isothermal, its use on-field would be possible using portable thermos-containers. DNA amplification was evaluated using calcein-manganese dye. Fluorescence at 320 nm indicated successful amplification. Samples treated with the conventional extraction kit and those treated following our in-house simplified methods, were all successfully amplified. Figure 6 illustrates LAMP outputs with the conventional extraction kit (Figure 6a) and with our best in-house method (MC, Figure 6b).

## 4. Discussion

Anthracnose is a serious, aggressive disease that attacks economically important crops every year. Yield losses in susceptible cultivars can reach up to 70–100% under conditions that propitiate the pathogen’s growth [8,14]. Rapid and correct diagnostic of the pathogens is undoubtedly crucial to minimizing and controlling outbreaks and limiting damage to the industry. Therefore, there is strong interest for the development of diagnostic tests suitable for the point-of-need applications. Gold nanoparticle-based immunochromatographic assays, using nitrocellulose membrane strips as the immunosorbent, have provided an unique analytical platform for the development of biosensors [54,55], without manipulating toxic reagents. Such paper-based microfluids are safe, disposable, self-sustainable, and allow for easy and rapid measurements. In addition, the confinement of the sample into the microfluidic environment of the device reduces the risk of sample contamination and has substantial reagent economy [56].

In the present study, we have successfully demonstrated that *C. gloeosp.* can be detected quickly, sensitively, and selectively using paper-based LFD. Our studies suggested that the LFD sensitivity could even be greater than conventional PCR as the device detected the pathogen in 100-fold diluted samples while PCR’s electropherogram only showed amplicons up to 10-fold dilution. The device was also selective to the target pathogen as test dots did not appear when using other pathogens such as *A. fum.* (different genus) and *C. theo.* and *C. cand.* (same genus). Even though nucleic acid amplification based tests or molecular diagnostics are very sensitive, selective, and reliable, their use in resource-limited settings, such as farms, may be constrained by scarce expertise and the need for expensive equipment [17,57]. In this study, those challenges have been addressed to assure the on-farm use of the fabricated dipstick.

**Figure 7:**
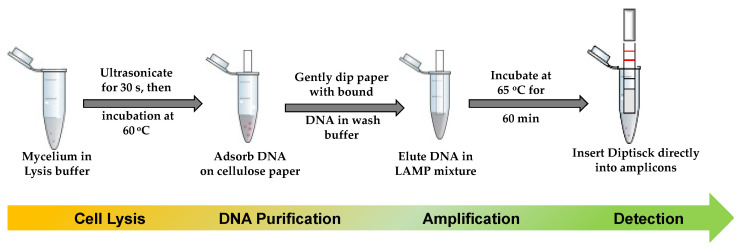
Summary diagram of a complete detection of *C. gloeosporioides* for on-farm use. The proposed methodology involving the solid-phase of nucleic acid extraction, an isothermal amplification, and the visual detection of the pathogen in just 5 quick steps. This protocol can be adaptable to different point-of-need uses and scalable to other pathogens.

LAMP has been presented as a low-cost genetic analysis tool for resource poor settings [58]. The method is said to be more tolerant to well-known PCR inhibitors and it is reported that an LAMP reaction can be implemented even when the DNA extraction step is eliminated [59,60]. While it is certain that there are a few outstanding challenges to enable LAMP reactions with crude sample materials, it is important to simplify the technique for full-scale usage in resource poor settings such as farms. For this purpose, we were able to successfully lyse and purify the fungal nucleic acid in just a few steps by using frequently used pre-combined chemicals, comparatively less than reported by Chi et al. [51] and Mason and Botella [53]. The use of ultrasonication, heat, and cellulose paper assure the need of minimal instruments adaptable for on-farm use, yet with the same efficiency if the sample is collected from the soil or directly from the leaves or fruits [50]. Extraction products where then amplified using LAMP. Yields of DNA molecules were not significantly different between each method, and qPCR had shown that all samples were successfully amplified, and positive LAMP reactions were also observed regardless of the extraction method. We selected Method C because it provided slightly higher DNA yields and better purity. As summarized in the Figure 7, our study confirmed that minimally instrumented/instrument-free molecular detection is possible for the diagnosis of *C. gloeosp*. In addition, the methodology presented in this work showed that *C. gloeosp.* can be detected quickly using LAMP technology.

While the visualization of fluorescence in LAMP is an important milestone, the integration of *C. gloeosp.*’s DNA extraction, LAMP amplification assays and LFD in a complete toolkit represents a highly promising approach for the development of point-of-care diagnosis/testing for anthracnose. In this line of research, similar diagnostic tools for clinical [61,62,63], food safety [23,64,65], and environmental monitoring [66,67] applications have already been studied. Most LFD only offer a qualitative YES/NO detection option of pathogens. Similarly, we have not provided color intensity values in this current work, since our current focus was a yes/no response. However, in the future as we shift focus to increasing detection sensitivity, we will analyze color intensity at test lines/dots. Nevertheless, few quantitative LFD biosensors [68] and low-cost 3D-printed antibody dispenser developments and small-scale production of lateral flow assays strips have been reported recently [69]. Further improvement including the evaluation of parameters important for on-field application, such as humidity and temperature with respect to stability of the immuno-dipstick, are ongoing and we hope to report the findings in the near future.

## 5. Conclusions

The current work described: (i) a fabricated immuno-dipstick for *C. gloeosporioides* (as far as we are aware, this is the first report); (ii) the specificity of the fabricated biosensor against closely related fungal species that also cause diseases to similar crops as our target fungus; (iii) simplified paper-based working DNA extraction protocol for *C. gloeosporioides*. Fungi have a strong cell wall. As such, the extraction of DNA from fungi is relatively more difficult than from bacteria, for example. The purity of the DNA and its subsequent amplification using LAMP (a sensitive-to-contamination but promising on-field technique) shows that the developed method is suitable for on-field (Figure 7). These proof-of-concept methods require integration steps to further simplify on-the-go pathogen detection. More research on low-cost technologies like portable sonicators, electronic battery-powered thermo containers [51], lyophilized chemicals for DNA extraction and LAMP reaction, will be crucial for fabricating a fully LAMP-integrated immuno-dipstick that is ideal and practical for point-of-need applications.

## Figures and Tables

**Table 1:** Primer Sequences.

Primer Name	Sequence (5′–3′)
Cg/f Int1	GACCCTCCCGGCCTCCCGCC
ITS4	TCCTCCGCTTATTGATATGC
ITS2	GCTGCGTTCTTCATCGATGC
ITS1	TCCGTAGGTGAACCTGCGG
FIP	GCCACTACCTTTGAGGGCCTACTTTCAACCCTCAAGCTCTGC
BIP	CGGAGCCTCCTTTGCGTAGTAAGGGTTTTACGGCAAGAGTCC
F3	ATGCCTGTTCGAGCGTC
B3	TCCGAGGTCAACCTTTGGAA
Cg/f Int1–Biotin	*biotin*—GACCCTCCCGGCCTCCCGCC
ITS4–FITC	*FITC*—TCCTCCGCTTATTGATATGC

## Supplementary Materials

The following are available online at https://www.mdpi.com/article/10.3390/bios12020049/s1, Figure S1: Dispersion and Stability evaluation of AuNPs conjugated with anti-biotin antibody based on the color, Table S1: Visual detection test of the immuno-dipstick based on different concentrations of AuNPs and different concentrations and volume of the immobilized antibody, Table S2: Quick and easy DNA extraction methods; reagents used and combined for each method.
