# Supplementary material for: Immuno-Dipstick for Colletotrichum gloeosporioides Detection: Towards On-Farm Application
Source: Biosensors (Basel). 2022 Jan 18;12(2):49. doi: 10.3390/bios12020049 (PMC8869205; doi:10.3390/bios12020049)
Supplement: Supplementary file 1 [file biosensors-12-00049-s001.zip › biosensors-1513580-supplementary.pdf]

Supplementary Materials:

Preparation and Optimization of AuNPs–Antibody Conjugates

The same initial concentration of AuNPs colloidal solution (Tubes A0, B0, and C0), anti-biotin antibody, prepared with different concentrations of the same buffer at the same pH was added. After an incubation period of 10 min at RT in a rotator, the color stability of tubes A1, B1, and C1 (corresponding to A0, B0, and C0, respectively) was checked. Tube C1 was the most stable, as it maintained its original red color. Lack of stability in the anti-biotin antibody–AuNPs conjugates could be observed as reflected by the sample solution changing from red to either purple, gray, clear, or precipitated. Last, the A1, B1, and C1 samples were kept for 8 days at RT to evaluate stability of the conjugates. Sample C2 maintained the red color while the others turned completely clear with black precipitates. Thus, sample C2, containing 0.02% (*w*/*v*) AuNPs and 50 μg/mL of anti-biotin antibody prepared with 5 mM KH_2PO_4 at pH 7.5, were considered the optimal conditions and used for subsequent experiments.

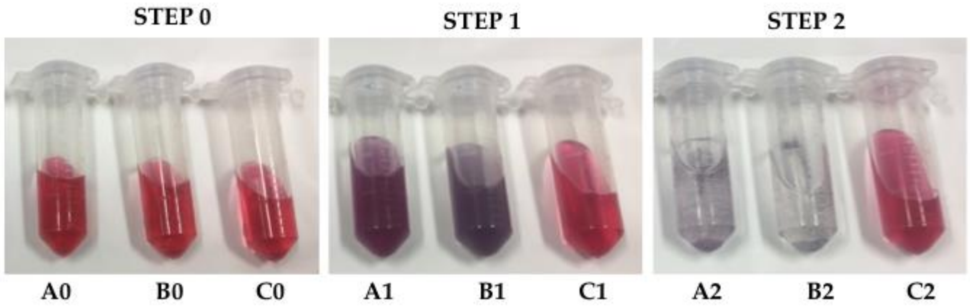

Figure S1. Dispersion and Stability evaluation of AuNPs conjugated with anti-biotin antibody based on the color.

As shown on the Table S2, the visual appearance of the control dot depended on several factors. The color of the control dot appeared better as the concentration of AuNPs used for the conjugation process increased. However, a high concentration and a low volume of the immobilized antibody (1mg/mL* 2 μL) gave a brighter dot with a clearer background.

Table S1. Visual detection test of the immuno-dipstick based on different concentrations of AuNPs and different concentrations and volume of the immobilized antibody.

Immobilized antibody concentration and volume	AuNPs colloidal solution concentrations (w/v) for conjugates preparations		
	0.0056 %	0.01%	0.02%
0.33 mg/mL *2 μl	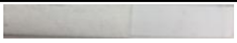	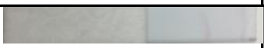	-
1 mg/ml*2 μl	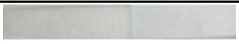	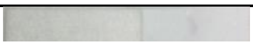	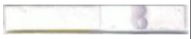
0.33 mg/ml *4 μl	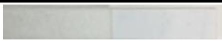	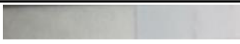	-
1 mg/ml*4 μl	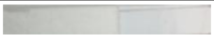	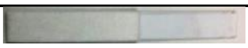	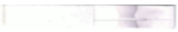

Table S2. Quick and easy DNA extraction methods; reagents used and combined for each method.

Method	Lysis buffer	Wash buffer
Method A	* Tris, 1 mM EDTA, 0.1 % SDS, 25 mM NaCl, 5 % (v/v) Tween-20	* Tris, 5 mM MgCl2
Method B	* Tris, 1 mM EDTA, 0.1 % SDS, 25 mM NaCl, 5 % (v/v) Tween-20	* Tris
Method C	* Tris, 1 mM EDTA, 0.1 % SDS, 25 mM NaCl	* Tris, 5 mM $MgCl_2$
Method D	* Tris , 1 mM EDTA, 0.1 % SDS, 25 mM NaCl	* Tris

*Tris = 10 mM Tris-HCl (pH 8.0)
